# Effects of a Whole Plant Extract of *Scutellaria rubropunctata* var. *rubropunctata* on Bone Metabolism with Estrogen Receptor Activation

**DOI:** 10.3390/plants11162075

**Published:** 2022-08-09

**Authors:** Misaki Watanabe, Tadahiro Yahagi, Takahiro Shirayama, Katsunori Miyake, Hitoshi Kotani, Takuya Ogawa, Keiichi Matsuzaki

**Affiliations:** 1 Laboratory of Pharmacognosy, School of Pharmacy, Nihon University, 7-7-1 Narashinodai, Funabashi 274-8555, Chiba, Japan; 2 School of Pharmacy, Tokyo University of Pharmacy and Life Sciences, 1432-1 Horinouchi, Hachioji 192-0392, Tokyo, Japan; 3 Faculty of Medicine, Shimane University, 1060 Nishikawatsu-cho, Matsue 690-8504, Shimane, Japan; 4 School of Pharmacy, International University of Health and Welfare, 2600-1 Kitakanemaru, Ohtawara 324-8501, Tochigi, Japan

**Keywords:** estrogen receptor, osteoporosis, osteogenesis, *Scutellaria rubropunctata* var. *rubropunctata*

## Abstract

We screened natural resources for estrogen receptor (ER)-activating and bone metabolism-promoting activities with the aim of finding potential treatments for osteoporosis. A screen of 1531 extracts from Ryukyu Arc plants using a luciferase reporter assay identified an 80% MeOH extract of *Scutellaria rubropunctata* var. *rubropunctata* (SRE) with dose-dependent ER transcription-promoting activity. Importantly, SRE had no proliferative effect on human breast cancer cells. SRE enhanced the ALP activity of pre-osteoblast MC3T3-E1 cells after 72 h in culture and slightly enhanced mineralization at 14 and 21 d. SRE did not significantly affect the TRAP activity of RAW264.7 cells. Gene expression analysis in MC3T3-E1 cells by quantitative real-time PCR revealed that SRE upregulated the mRNA levels of *Runx2*, *Osterix* (*Osx*), *Osteopontin* (*Opn*), *Osteocalcin* (*Ocn*), *Smad1*, *Smad4*, and *Smad5* at 72 h, and those of *Runx2*, *Osx*, *Smad1*, *Smad4*, and *Smad5* at 21 d of osteogenic induction. Analysis of the expression levels of osteogenic markers suggested that SRE may promote osteogenic differentiation by acting at the early stage of differentiation rather than at the late stage of differentiation. These results indicate that SRE activates ER and induces osteoblast differentiation by activating *Runx2* and *Osx* through the BMP/*Smad* pathway, suggesting that SRE may be useful for the prevention and treatment of postmenopausal osteoporosis.

## 1. Introduction

Estrogen secretion rapidly declines in women in their late 40s and decreases even further after menopause as ovarian function declines [1]. This decline can cause hot flashes, mood swings, urogenital atrophy, and other unpleasant symptoms. Estrogen-decline-related onset of postmenopausal osteoporosis due to bone loss causes chronic pain, bone deformity, and increased susceptibility to severe fractures, and is considered one of the most significant causes of reduced quality of life and mobility in postmenopausal women [2,3].

According to the WHO diagnostic classification, osteoporosis causes decreased bone mass and bone mineral density and deterioration of the bone structure, leading to an increased risk of fracture. Bone is maintained via bone formation by osteoblasts and bone resorption by osteoclasts. Estrogen regulates bone resorption through estrogen receptors α (ERα) and β (ERβ) by acting directly and indirectly on osteoclasts, is involved in the proliferation of osteoblast precursor cells and mesenchymal stem cells, and helps maintain bone tissue and matrix balance by inducing the expression of TGF-β [4,5,6,7,8,9]. Estrogen depletion can be treated with hormone replacement therapy (HRT), which alleviates symptoms in menopausal women and reduces osteoporosis [10]. However, long-term HRT is known to increase the risk of breast cancer and other cancers [10,11]. Therefore, selective estrogen receptor modulators (SERMs) with tissue-selective estrogen action have recently attracted attention for the treatment of postmenopausal osteoporosis, as they have minimal effect on reproductive organs.

Some plants and natural bioactive compounds exhibit SERM-like effects, such as phytoestrogens [12]. For example, isoflavones, such as daidzein and genistein, phytoestrogens in soybeans, and puerarin in the root of *Pueraria lobata*, have been used as supplements and in health foods for their estrogenic effects to promote bone mass maintenance and relieve menopausal symptoms, including osteoporosis, in postmenopausal women [13,14,15]. Therefore, natural resources that activate ER and affect bone metabolism may be useful for the treatment of postmenopausal osteoporosis. The genus *Scutellaria*, in the family *Lamiaceae*, comprises 17 species native to Japan [16]. *S*. *rubropunctata* var. *rubropunctata* (SR) is a plant that grows naturally only in the region known as the Ryukyu Arc, which includes the Amami Oshima and Okinawa Islands. However, there have been no reports on its constituents and their bioactivity.

In this study, we screened a library of 1531 extracts of Ryukyu Arc plants (10 µg/mL) and detected ER transcription activity in an 80% MeOH extract of the whole plant of SR (SRE). We then examined its effects on bone metabolism in vitro using osteoblasts and osteoclasts.

## 2. Results

### 2.1. SRE Has ER Transcription-Promoting Activity

**Figure 1:**
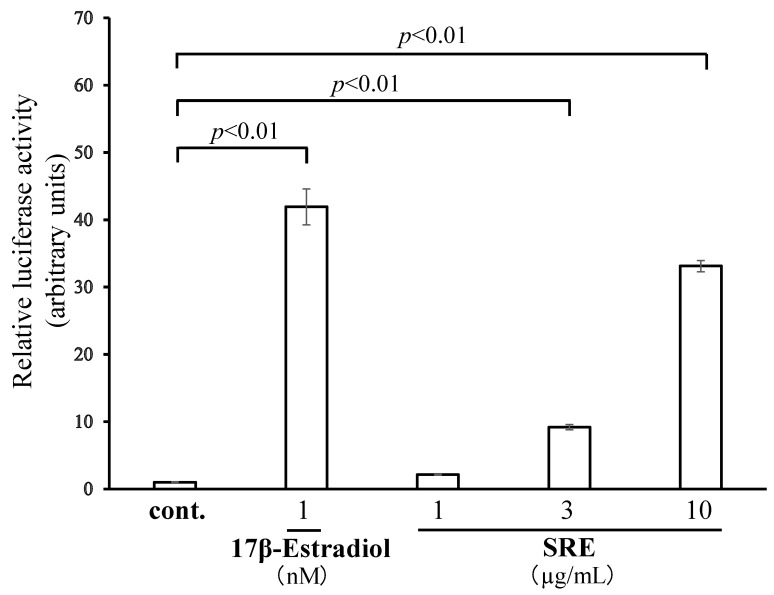
Estrogen receptor (ER) transcription-promoting activity of an 80% MeOH extract of *Scutellaria rubropunctata* var. *rubropunctata* (SRE). The ER transcription-promoting activity of SRE was examined using a luciferase reporter assay in HEK293 cells. The control (cont.), which contained vehicle only (DMSO), was set to 1. Data are the mean ± SE of three independent experiments (*n* = 3).

**Figure 2:**
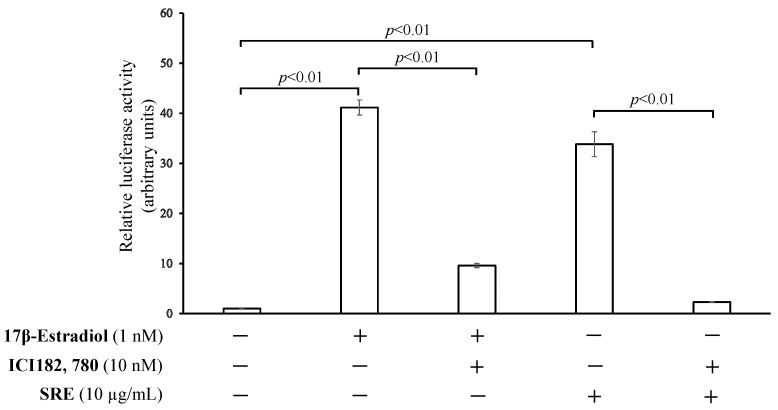
Effect of the ER antagonist ICI 182, 780 on the ER transcription-promoting activity of SRE. The activity was assessed using a luciferase reporter assay in HEK293 cells. The control (first column) contained vehicle only (DMSO). Data are the mean ± SE of three independent experiments (*n* = 3).

The ER-activating effect of SRE (1–10 µg/mL) was examined using a luciferase reporter assay in HEK293 cells. SRE, at 3 and 10 µg/mL, significantly increased ER transcription activity (*p* < 0.01; Figure 1). To confirm that activation in the reporter assay was mediated by ER, we conducted the assay using the concentration of SRE with the strongest ER transcription-promoting activity (10 µg/mL) and an ER antagonist (ICI182, 780). In this assay, the ER activation effect of SRE was significantly inhibited by ICI182, 780 (Figure 2), suggesting that SRE activates ER.

### 2.2. SRE Does Not Affect MCF7 Cell Proliferation

**Figure 3:**
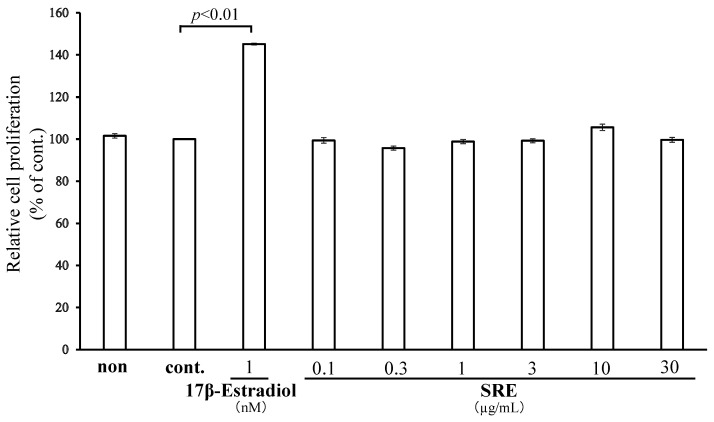
SRE has no proliferation-promoting effect on human breast carcinoma cells. The proliferation of MCF7 cells was assessed using the MTT assay. The control contained DMSO only. Data are the mean ± SE of three independent experiments (*n* = 3).

The effect of SRE (0.1–30 µg/mL) on the growth of the MCF7 breast cancer cell line was evaluated using the MTT assay. No significant proliferation was detected at any tested concentration, suggesting that SRE has estrogen-like effects but no proliferation-promoting effects on breast carcinoma cells (Figure 3).

### 2.3. Effect of SRE on Osteoblastic Differentiation

To determine the effects of SRE on the early and late stages of osteoblastic differentiation, an alkaline phosphatase (ALP) activity assay and mineralization staining were performed in MC3T3-E1 osteoblast-like cells. Its effects on genes involved in osteoblast differentiation were examined using quantitative real-time PCR.

#### 2.3.1. SRE Increased ALP Activity and the mRNA Expression of *Alpl*

**Figure 4:**
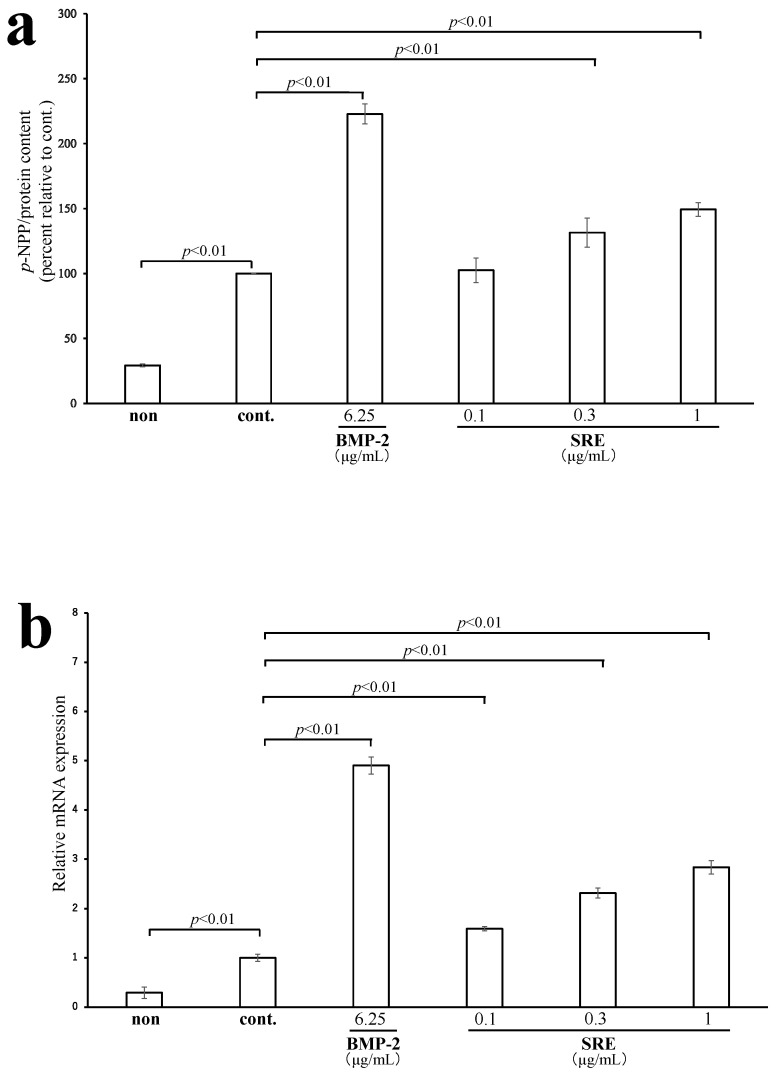
Effect of SRE on (**a**) alkaline phosphatase (ALP) activity and (**b**) the mRNA expression level of *Alpl* in the MC3T3-E1 mouse osteoblastic cell line. The “non” sample was incubated without differentiation inducer but with vehicle (DMSO), and the control (cont.) was incubated with a differentiation medium and DMSO. The mRNA expression level of *Alpl* was measured by quantitative real-time PCR and normalized to the level of the reference gene *β-actin*. Data are the mean ± SE of three independent experiments (*n* = 3).

MC3T3-E1 cells were incubated with SRE (0.1–1 µg/mL) in the presence of differentiation inducers, and ALP activity and the mRNA expression level of *Alpl* were assessed. Significant ALP activity was observed in the presence of 0.3 and 1 µg/mL SRE (*p* < 0.01; Figure 4a) without cytotoxicity (Figure S1). In addition, SRE significantly increased the mRNA expression level of *Alpl* (*p* < 0.01; Figure 4b) in a dose-dependent manner, suggesting that SRE may promote the early stage of osteoblast differentiation.

#### 2.3.2. SRE Increased Mineralization

**Figure 5:**
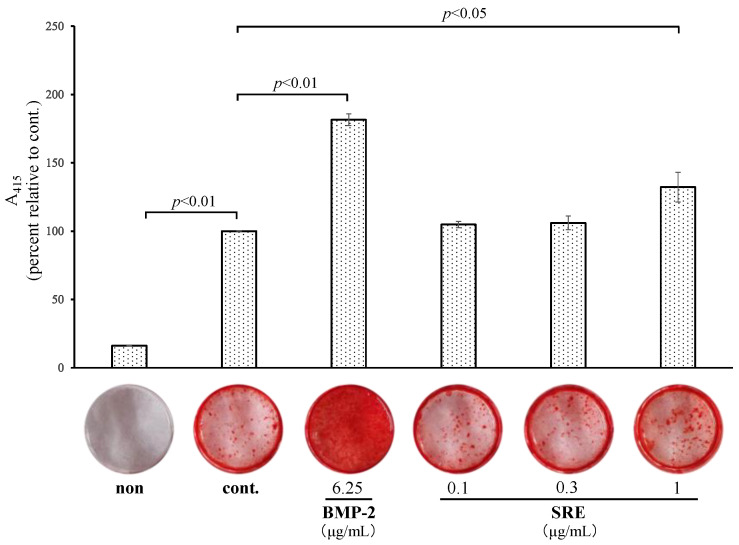
Effect of SRE on mineralization in MC3T3-E1 cells incubated for 14 d. The “non” sample was incubated in a growth medium with DMSO, and “cont.” was incubated in a differentiation medium with DMSO. Data are the mean ± SE of three independent experiments (*n* = 3).

**Figure 6:**
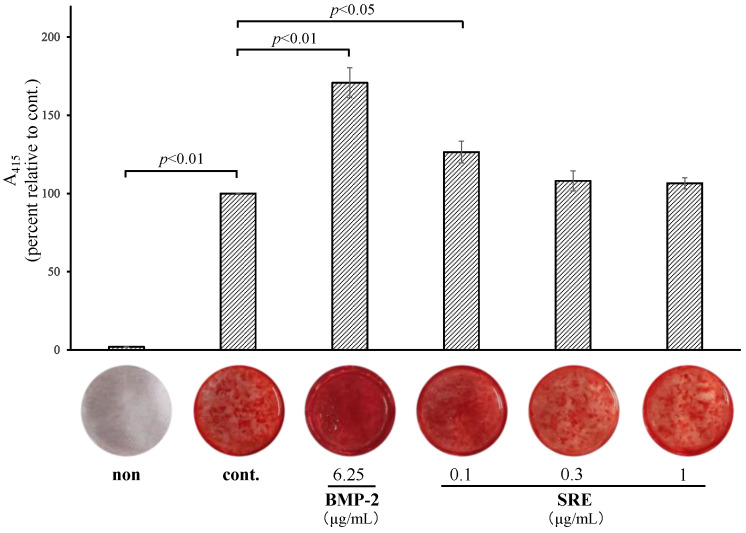
Effect of SRE on mineralization in MC3T3-E1 cells incubated for 21 d. The “non” sample was incubated in a growth medium with DMSO, and the cont. sample was incubated in a differentiation medium with DMSO. Data are the mean ± SE of three independent experiments (*n* = 3).

The effect of SRE on mineralization in MC3T3-E1 cells in long-term culture (14 or 21 d) with differentiation inducers was examined. The cells were incubated with differentiation inducers and various concentrations of SRE. SRE significantly enhanced mineralization at 14 d (1 µg/mL, *p* < 0.05; Figure 5) and 21 d (0.1 µg/mL, *p* < 0.05; Figure 6). However, the effect of SRE was lower than that of the positive control, BMP-2. These results suggest that SRE may have a weak effect on the late stage of osteoblast differentiation.

#### 2.3.3. Effect of SRE on the Expression of Osteoblast-Differentiation-Related Genes

**Figure 7:**
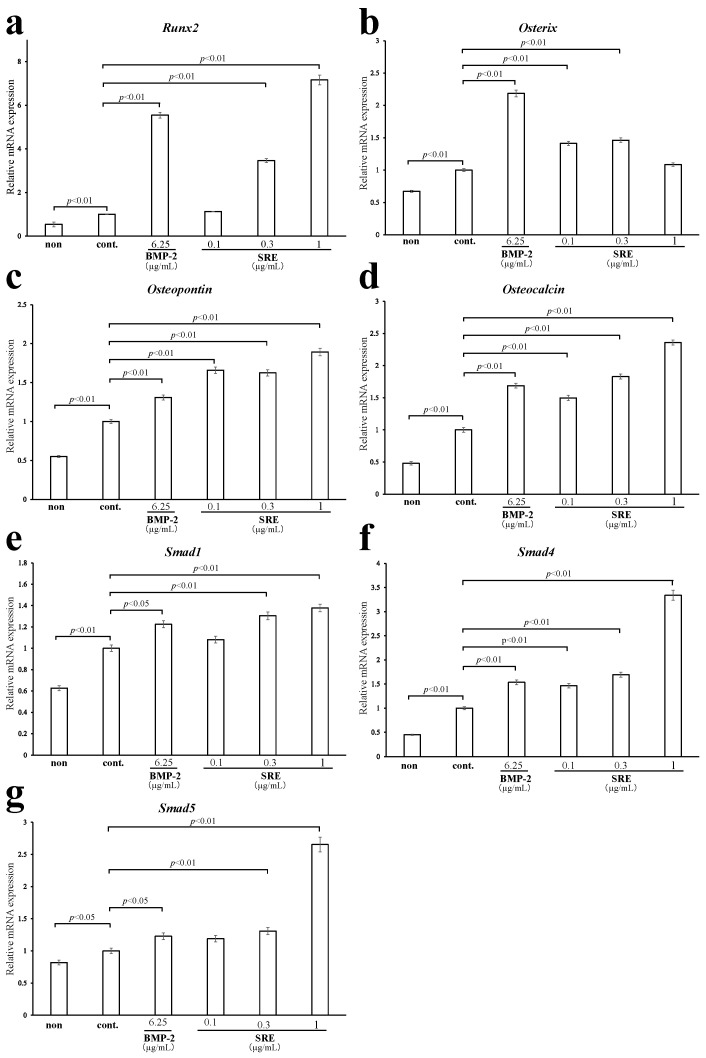
Effect of incubation with SRE for 72 h on the mRNA expression of osteoblast-differentiation-related genes in MC3T3-E1 cells. The mRNA expression levels of (**a**) *Runx2*, (**b**) *Osterix*, (**c**) *Osteopontin*, (**d**) *Osteocalcin*, (**e**) *Smad1*, (**f**) *Smad4*, and (**g**) *Smad5* were measured by quantitative real-time PCR and normalized to the levels of the reference gene *β-actin*. The “non” sample was incubated in an ordinary medium with DMSO, and the “cont.” sample” was incubated in a differentiation medium with DMSO. Data are the mean ± SE of three independent experiments (*n* = 3).

**Figure 8:**
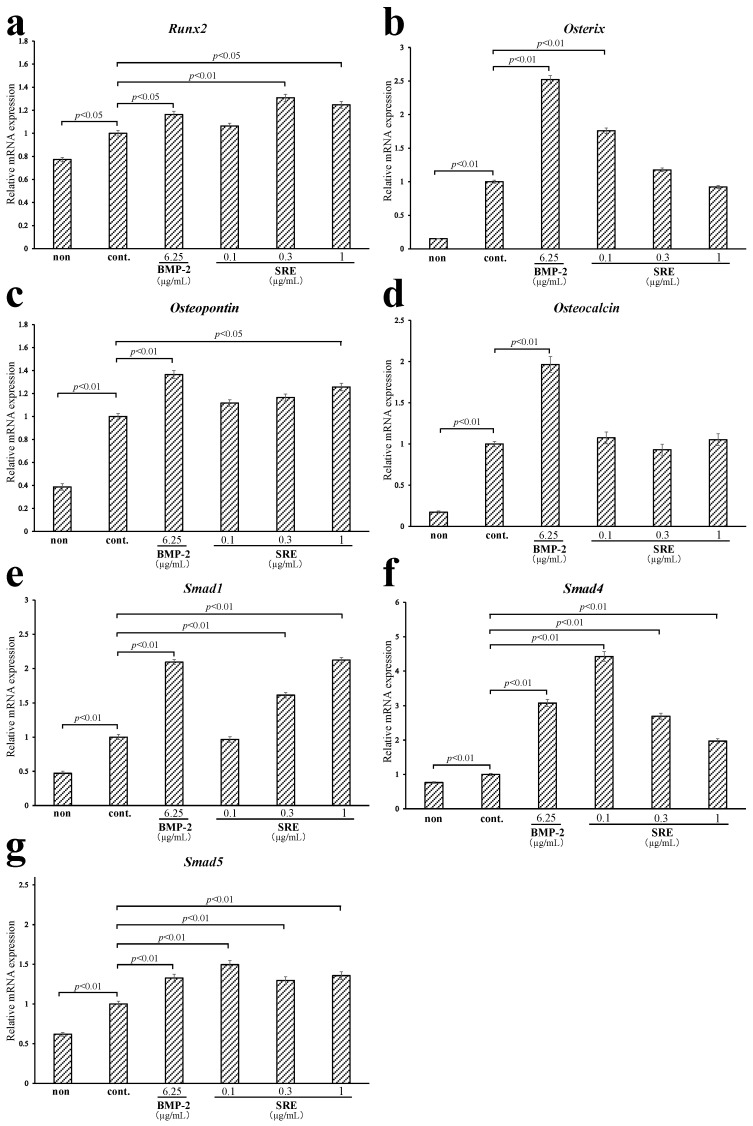
Effect of incubation with SRE for 21 d on the mRNA expression of osteoblast-differentiation-related genes in MC3T3-E1 cells. The mRNA expression levels of (**a**) *Runx2*, (**b**) *Osterix*, (**c**) *Osteopontin*, (**d**) *Osteocalcin*, (**e**) *Smad1*, (**f**) *Smad4*, and (**g**) *Smad5* were measured by quantitative real-time PCR and normalized to the levels of the reference gene *β-actin*. The “non” sample was incubated in an ordinary medium with DMSO, and the “cont.” sample was incubated in a differentiation medium with DMSO. Data are the mean ± SE of three independent experiments (*n* = 3).

To elucidate the mechanism underlying the osteoblastic differentiation-promoting effect of SRE at the gene expression level, we used quantitative real-time PCR to examine the expression of osteoblast-differentiation-related genes in MC3T3-E1 cells incubated in a differentiation medium with different concentrations of SRE (0.1–1 µg/mL) for 72 h or 21 d. SRE significantly increased the expression levels of *Runx2*, a master regulator of bone formation, at 72 h (0.3 and 1 µg/mL, *p* < 0.01) and 21 d (0.3 and 1 µg/mL, *p* < 0.01 and *p* < 0.05; Figure 7a and Figure 8a). The expression levels were comparable to those in cells incubated with BMP-2. These results indicate that SRE may promote the early stage of osteoblast differentiation, which is similar to the results of the ALP activity assay. Since *Runx2* promotes the early stage of osteogenesis but inhibits the late stage when osteoblasts mature, SRE may attenuate bone formation in the late stage [17].

*Osterix* is an essential transcription factor for osteoblast differentiation and bone formation that is downstream of *Runx2*. SRE significantly upregulated the expression levels of *Osx* mRNA at 72 h (0.1 and 0.3 µg/mL, *p* < 0.01) and 21 d (0.1 µg/mL, *p* < 0.01; Figure 7b and Figure 8b), but SRE-promoted *Osx* expression levels were lower than the BMP-2-promoted levels at 72 h and 21 d. These data show that SRE induces osteoblast differentiation via transcriptional activation of *Osx*, but the effect is not as strong as that of BMP-2.

*Osteopontin* is a target gene of *Runx2* and *Osterix,* and is a bone formation marker that is produced during early-to-mid-stage osteoblastic differentiation [18]. SRE significantly elevated the mRNA expression of *Osteopontin* at both 72 h (0.1–1 µg/mL, *p* < 0.01) and 21 d (1 µg/mL, *p* < 0.05; Figure 7c and Figure 8c). *Osteopontin* expression levels increased more in the presence of SRE than in the presence of BMP-2 at 72 h, but increased less in the presence of SRE than in the presence of BMP-2 at 21 d. These results indicate that SRE may promote osteoblast differentiation in the early stage and may have a weaker effect in the later stages. SRE significantly increased the mRNA expression levels of *Osteocalcin* at 72 h in a dose-dependent manner (0.1–1 µg/mL, *p* < 0.01), which was higher that the expression induced by BMP-2 (Figure 7d). In contrast, SRE did not affect the mRNA expression of *Osteocalcin* at 21 d (Figure 8d). *Osteocalcin* is also a target gene of *Runx2* and *Osterix* and is a bone formation marker produced during the early-to-mid-stage of osteoblastic differentiation [18]. These results support the hypothesis that SRE promotes osteoblast differentiation in the early stage and weakly promotes late-stage differentiation.

One of the signaling pathways that induces the expression of *Runx2* and *Osterix* is the BMP/*Smad* signaling pathway. Therefore, we assessed the mRNA expression levels of *Smad1*, *Smad4*, and *Smad5*, three transduction factors that promote bone formation, using quantitative real-time PCR to determine whether the osteoblastic differentiation effect of SRE extract was related to this signaling pathway. SRE significantly enhanced the mRNA expression levels of *Smad1* at 72 h and 21 d (0.1 and 0.3 µg/mL, *p* < 0.01; Figure 7e and Figure 8e), and the expression levels were close to those induced by BMP-2 at both time points. SRE significantly elevated the mRNA expression of *Smad4* at 72 h and 21 d (0.1–1 µg/mL, *p* < 0.01; Figure 7f and Figure 8f), and the expression levels were as high as or higher than those induced by BMP-2. SRE significantly increased the mRNA expression of *Smad5* at 72 h (0.3 and 1 µg/mL, *p* < 0.01) and 21 d (0.1–1 µg/mL, *p* < 0.05; Figure 7g and Figure 8g). The *Smad5* expression levels were as high or higher than those induced BMP-2 at both 72 h and 21 d. These results, showing that SRE increased the mRNA expression levels of *Smads 1*, *4*, and *5*, indicate that SRE may promote bone formation via the BMP/*Smad* signaling pathway.

### 2.4. Effect of SRE on Osteoclastic Differentiation

**Figure 9:**
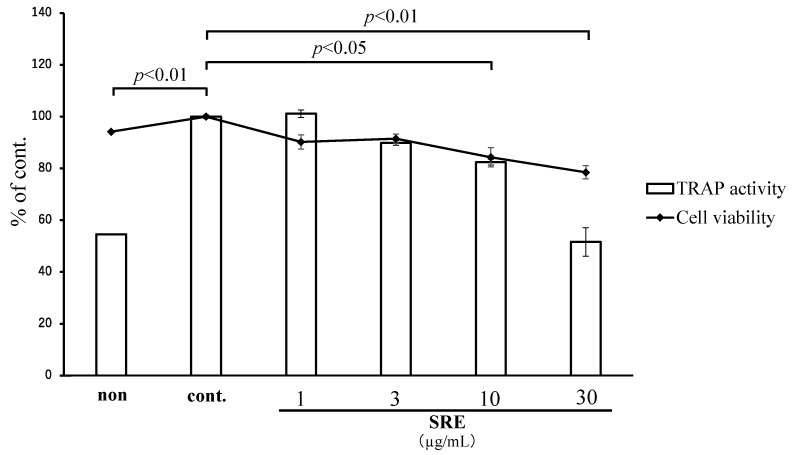
Effect of SRE on tartrate-resistant acid phosphatase (TRAP) activity and viability in osteoclastic cells. The “non” sample was incubated in a growth medium with DMSO, and the “cont.” sample was incubated with sRANKL and DMSO. Cell viability was measured by the MTT assay. Data are the mean ± SE of three independent experiments (*n* = 3).

To evaluate the effect of SRE on bone resorption, a tartrate-resistant acid phosphatase (TRAP) activity assay was performed on osteoclast-like cells (RAW264.7 cells differentiated with RANKL). TRAP activity was significantly suppressed by SRE at 10 and 30 µg/mL (*p* < 0.05 and *p* < 0.01; Figure 9); however, it was also cytotoxic at these concentrations. This result suggests that the inhibitory effect of SRE on osteoclastic differentiation may be caused by cytotoxic effects at these concentrations.

## 3. Discussion

We aimed to identify natural products that help prevent and treat postmenopausal osteoporosis by stimulating estrogen receptors (ER) and promoting osteogenesis. A screening of 1531 extracts of Ryukyu Arc plants using a luciferase reporter assay in HEK293 cell showed that SRE had dose-dependent ER transcription-promoting activity. The effect of SRE was significantly inhibited by the ER antagonist ICI182, 780, suggesting that SRE activates ER. Substances exhibiting estrogen-like effects often exert proliferative effects on estrogen-sensitive cancers, such as breast carcinoma [10,11]. Therefore, we tested SRE for growth-promoting effects on MCF7 breast cancer cells, and no significant cell proliferation was observed. These results show that SRE may selectively stimulate ER. Next, we evaluated the effects of SRE on bone metabolism using the MC3T3-E1 osteoblast-like cell and RAW264.7 cell induced to differentiate with RANKL, which is an osteoclast-like cell.

Osteoblasts differentiate from mesenchymal stem cells in a stepwise manner into osteoprogenitor cells, pre-osteoblasts, and osteoblasts, which eventually become osteocytes through mineralization [19]. Bone-specific ALP, a biochemical marker of bone formation, is an enzyme that converts pyrophosphate to phosphoric acid on the surface of matrix vesicles released from osteoblast [19,20]. Pyrophosphate is a substrate for ALP and is an inhibitory factor in the crystallization of calcium and phosphorus. After conversion of pyrophosphate to phosphoric acid by ALP, the minerals crystallize to hydroxyapatite, which penetrates matrix vesicles and is deposited in the bone matrix, promoting bone mineralization [21]. SRE significantly upregulated ALP activity and the mRNA expression of *Alpl* in MC3T3-E1 cells, suggesting it has a positive effect on the differentiation of osteoprogenitor and pre-osteoblast cells into osteoblasts. When MC3T3-E1 cells were cultured in the presence of differentiation inducers, they began to form calcified nodules after about 14 d, and the matrices matured after about 21 d [22]. To examine the effect of SRE at these time points, cells were stained alizarin red, which showed that SRE increased the mineralization rate at 14 and 21 d, but its influence on mineralization was weaker than that of the positive control, BMP-2. Therefore, SRE may have a slight promoting effect at the late stage of osteoblast differentiation.

Next, the effects of SRE on the expression of osteogenic-related genes were also analyzed using quantitative real-time PCR. *Runx2* is a master regulator of osteogenesis that induces the differentiation of immature osteoblasts. In contrast, *Runx2* suppresses the differentiation of mature osteoblasts and is generally assumed to prevent bone hyperplasia [17]. *Osterix*, which acts downstream of *Runx2*, is an essential transcription factor for bone formation that contributes at all stages of differentiation. These transcription factors promote the production of enzymes and non-collagenous proteins, such as ALP, Osteopontin, and Osteocalcin, which are bone formation markers [23]. The mRNA expression levels of *Runx2*, *Osx*, *Opn*, and *Ocn* after 72 h of differentiation induction were significantly induced by treatment with SRE. These data support the hypothesis that SRE promotes the early stage of osteoblast differentiation. After 21 d of differentiation culture, SRE increased the mRNA expression levels of *Osx* and *Opn* at low concentration, but the expression levels were less than those induced by BMP-2. In addition, SRE did not affect the mRNA expression of Osteocalcin. These expression analyses and alizarin red staining data suggest that SRE may promote late-stage osteogenesis to a slight degree. The cause of the slight effect of SRE on mature osteoblasts might be related to its weaker induction of *Osx* mRNA expression than that attributable to BMP-2, even though the extract induced mRNA expression in *Runx2*, an inhibitor of mature osteoblasts, to similar levels as BMP-2. In the alizarin red staining assay, the concentration that best promoted mineralization shifted from 1 µg/mL at 14 d to 0.1 µg/mL at 21 d. One possible reason for this phenomenon is that the function of *Runx2* changes as differentiation progresses. SRE increased *Runx2* mRNA expression in the early and late stages of osteoblast differentiation; therefore, the extract might significantly promote osteoblast differentiation until calcified nodules begin to form at 14 d and then change to slight promotion of osteoblast differentiation until matrix maturation at 21 d.

One of the intracellular signal pathways that activates the transcription of *Runx2* and *Osterix* is the BMP/*Smad* signaling pathway. BMP-2 is related to the production of bone matrix proteins that turn on three cytoplasmic proteins, *Smads 1*, *5*, and *8*, via BMP receptors, and the activated *Smads 1*/*5*/*8* form a complex with *Smad4*. After the complexes translocate into the nucleus, they bind to *Smad*-binding elements, which are in the transcriptional regulatory domain and regulate the transcription of *Runx2* [24]. *Smads* are classified into three groups: receptor-regulated *Smads* (R-*Smads*), common-partner *Smads* (Co-*Smads*), and inhibitory *Smads* (I-*Smads*). *Smads 1* and *5* are R-*Smads* that specifically transduce BMP signaling, and *Smad4* is a Co-*Smad* involved in signaling transduction by forming a complex with R-*Smads* [25]. SRE increased the mRNA expression levels of *Smads 1*, *4*, and *5* at 72 h and 21 d of culture, indicating that *Smads 1*, *4*, and *5* were activated from the early phase through the late stage, and SRE may promote osteogenesis via BMP/*Smad* signaling. 17β-Estradiol promotes osteoblastic differentiation but does not affect the mRNA expression of *Runx2*. Thus, the effect of SRE on MC3T3-E1 cells may be mediated by activation of the BMP/*Smad* signaling pathway rather than by ER ligand activity [26,27].

When monocytic and macrophagic cells are stimulated by the receptor activator of NF-κB ligand (RANKL), they differentiate into mononuclear osteoclasts, which fuse with multinuclear osteoclasts that have bone resorption ability [28]. Tartrate-resistant acid phosphatase (TRAP) is an enzyme produced when progenitor cells differentiate into osteoclasts and is a marker of bone resorption [29]. SRE significantly inhibited TRAP activity but also had a cytopathic effect. Therefore, suppression of TRAP activity may be caused by cytotoxicity, and SRE may have no specific effect on bone resorption. This lack of a bone resorption effect may seem to be a negative for osteoporosis treatment; however, strong bone resorption inhibitors, such as bisphosphonates, may increase the risk of atypical femur fractures resulting from abnormal bone remodeling [30,31]. Consequently, we consider agents that promote bone formation without inhibiting bone resorption could improve osteoporosis while maintaining normal bone remodeling.

To improve menopausal symptoms caused by low estrogen production, such as hot flashes and depression, many people eat foods or take supplements with mild estrogen-like effects, such as *Vitex trifolia* L., rather than seeking treatment at a hospital [32,33]. In addition to the aforementioned unpleasant symptoms, dramatic reductions in bone mass and density are a concern, but few supplements or foods provide both menopausal symptom relief and bone maintenance benefits.

SERMs, the first-line treatment for postmenopausal osteoporosis, have side effects related to low estrogen symptoms, such as hot flashes and leg cramps. Therefore, clinical trials have recently been conducted combining SERMs and estrogen preparations in a treatment called tissue selective estrogen complex (TSEC) [34,35,36,37,38,39]. TSEC increased bone mineral density in the lumbar spine and the femoral neck, improved lipid profiles, and ameliorated hot flashes and vaginal conditions better than raloxifene or bazedoxifene alone [35,39]. It is possible that SRE, which not only has estrogen-like effects but also exerts bone-forming effects independent of ER, may improve both postmenopausal symptoms and osteoporosis risk and improve quality of life during menopause.

SR is an underutilized plant that has never been studied, and its constituents are still unknown. A large number of flavonoids, such as baicalin and wogonin, have been reported as constituents of the root of *S. baicalensis* Georgi (SB) [40], a plant of the same genus. HPLC analysis of SRE was carried out under conditions that allowed analysis of compounds of a wide range of polarity in SB extract (SBE). HPLC chromatograms of SRE (Figure S2) showed compounds with absorption similar to flavonoids such as baicalin and wogonin in SBE. It also showed peaks with maximum absorption around 250–270 nm and 300–350 nm (*t*_R_: 6.14, 6.49, 7.34, 13.25 and 13.49 min) in the wide range of polarity, suggesting that it may contain flavonoids as a major component.

## 4. Materials and Methods

### 4.1. Plant Material and Extract Preparation

The whole plant of SR was collected on Amami-Oshima (Kagoshima, Japan) in August 2019 and was identified by a taxonomist (Nobuyuki Tanaka). Voucher specimens were deposited in the School of Pharmacy, Tokyo University of Pharmacy and Life Sciences (E08269) and the TNS herbarium (TNS0134184). SR was dried before extraction. SB was purchased from Uchida Wakanyaku Ltd. (Tokyo, Japan). SR and SB (1 g each) were pulverized, then extracted with 80% MeOH aq. (10 mL) by sonication (15 min) and concentrated under low pressure. The obtained SRE was dissolved in DMSO (Kanto Chemical, Tokyo, Japan) for use in subsequent experiments.

### 4.2. Cell Lines and Cell Culture

HEK293 human embryonic kidney cells, MC3T3-E1 mouse calvaria-derived pre-osteoblastic cells, RAW264.7 mouse macrophage-like cells, and MCF7 human breast carcinoma cells were obtained from the RIKEN BioResource Research Center (Ibaraki, Japan). HEK293 and MCF7 cells were cultured in Dulbecco’s Modified Eagle’s Medium (DMEM; Nacalai Tesque, Kyoto, Japan), supplemented with 10% fetal bovine serum (FBS; Thermo Fisher Scientific, Waltham, MA, USA) and 1% penicillin/streptomycin (P/S; Nacalai Tesque). MC3T3-E1 and RAW264.7 cells were cultured in α-minimum essential medium (α-MEM; Nacalai Tesque) supplemented with 10% FBS and 1% P/S. All cultures were incubated at 37 °C with 5% CO_2_.

### 4.3. Luciferase Reporter Assays of ER Transcription Activity

HEK293 cells grown to 70% confluence were transfected with the expression plasmid pBIND-ERα (50 ng/μL; Promega, Madison, WI, USA) and reporter plasmid pG5-Luc (50 ng/μL; Promega) using PEI MAX (50 µg/mL; Polysciences, Warrington, PA, USA) and Opti-MEM (Thermo Fisher Scientific). After incubation of the plasmids with the transfection reagent for 20 min at room temperature, HEK293 cells were transfected with the plasmid-reagent solution for 6 h at 37 °C. Transfected cells were seeded in a 96-well plate at 2 × 10^4^ cells/well and treated with screening sample (10 µg/mL) or various concentrations of SRE or 1 nM 17β-estradiol (positive control; Sigma-Aldrich, St. Louis, MO, USA) in DMSO at 37 °C for 24 h. After incubation, the medium was then removed, and luminescence was measured using a luciferase assay system (Promega) with a FLUOstar Omega microplate reader (BMG LABTECH, Ortenberg, Germany) according to the manufacturer’s instructions.

### 4.4. ALP Activity Assay

MC3T3-E1 cells were seeded in a 24-well plate at a density of 1 × 10^5^ cells/well. After the cells reached 90% confluence, the culture medium was exchanged with differentiation medium containing Osteoblast-Inducer Reagent (TaKaRa Bio, Shiga, Japan). Then, the cells were incubated with various concentrations of SRE or 6.25 µg/mL bone morphogenetic protein-2 (BMP-2; Bio Legend, CA, USA) for 72 h. Cell lysates were obtained by adding 1% NP-40 (Nacalai Tesque) and were diluted with buffer [0.2 M Tris-HCl (pH 9.5) and 1 mM MgCl_2_]. ALP activity was measured using the LabAssay ALP Kit (FUJIFILM Wako Pure Chemical, Tokyo, Japan) according to the manufacturer’s instructions. The absorbance at 405 nm was measured using a FLUOstar Omega microplate reader and was compared with the absorbance of *p*-nitrophenol standards. The protein concentration of the cell lysates was determined using the TaKaRa BCA Protein Assay Kit (TaKaRa Bio). The results are expressed as the concentration of *p*-nitrophenol per µg of protein as a ratio (%) of the control.

### 4.5. Mineralization Assay

MC3T3-E1 pre-osteoblast cells were seeded on a 24-well plate at a density of 1 × 10^5^ cells/well and grown until 90% confluence. The culture medium was then changed to differentiation medium, and the cells were exposed to various concentrations of SRE or 6.25 µg/mL BMP-2 (positive control). The medium was changed every 3–4 d. After 14 and 21 d of differentiation, the supernatant was removed, and the cells were fixed with 10% formaldehyde neutral buffer (Nacalai Tesque). The fixed cells were stained with alizarin red solution (IWAI CHEMICALS, Tokyo, Japan) according to the manufacturer’s instructions, and 5% formic acid was added to each well. The absorbance at 415 nm of the lysates was measured using a FLUOstar Omega microplate reader.

### 4.6. TRAP Activity Assay

RAW264.7 cells were seeded in a 96-well plate at a density of 5 × 10^3^ cells/well and incubated for 24 h at 37 °C. Then, differentiation to osteoclasts was induced by adding a recombinant receptor activator of NF-κB ligand (sRANKL; Oriental Yeast Co., Tokyo, Japan). The cells were treated with various concentrations of SRE at the same time. After incubation for 5 d at 37 °C, the supernatant was removed, and the differentiated cells were lysed and incubated in 50 mM citrate buffer (pH 4.6) containing 10 mM tartrate, 0.1% TritonX-100, and 10 mM *p*-nitrophenyl phosphate hexahydrate for 30 min at 37 °C. The reaction was terminated by the addition of 2 mM NaOH, and then the absorbance was measured at 405 nm.

### 4.7. Measurement of Cell Proliferation by the MTT Assay

MCF7 cells and RAW264.7 cells were plated in a 96-well plate at a density of 1 × 10^5^ cells/mL and incubated for 24 h at 37 °C. Then the medium was changed, and the cells were incubated with various concentrations of SRE for 48 h or 5 d. After incubation, 0.1 mg/mL MTT was added to each well and incubated at 37 °C for 4 h. The supernatant was removed, and DMSO was added to each well to dissolve the formazan crystals. The absorbance was measured with a microplate reader at a wavelength of 570 nm and a reference wavelength of 655 nm.

### 4.8. Quantitative Real-Time PCR

Total RNA was extracted from MC3T3-E1 cells that were differentiated as described in the ALP and mineralization assays using the RNeasy mini kit (Qiagen, Valencia, CA, USA) according to the manufacturer’s instructions. cDNA was synthesized from 250 ng of total RNA using the Prime Script RT-PCR kit (Qiagen) and a TaKaRa PCR Thermal Cycler Dice (TaKaRa Bio). Quantitative real-time PCR was performed using GoTaq^®^ Green Master Mix (Promega) according to the manufacturer’s instructions on an Applied Biosystems^®^ StepOnePlus™ (Thermo Fisher Scientific). The cycling conditions were 95 °C for 2 min, followed by 40 cycles of denaturation at 95 °C for 15 s and annealing and extension at 65 °C for 1 min. The mRNA expression levels of the target genes were calculated by the 2^−ΔΔCt^ method using StepOne Software v2.3, and were normalized to *β-actin*. The primers used were as follows: *Alpl* sense: 5′-GCA GTA TGA ATT GAA TCG GAA CAA C-3′ and antisense: 5′-ATG GCC TGG TCC ATC TCC AC-3′, *Runx2* sense: 5′-AGG GAC TAT GGC GTC AAA CA-3′ and antisense: 5′-GGC TCA CGT CGC TCA TCT T-3′, *Osx* sense: 5′-CGC TTT GTG CCT TTG AAA T-3′ and antisense: 5′-CCG TCA ACG TTA TGC-3′, *Ocn* sense: 5′-CAG ACA AGT CCC ACA CAG-3′ and antisense: 5′-GCA GAG TGA GCA GAA AGA-3′, *Opn* sense: 5′-ACA CTT TCA CTC CAA TCG TCC CTA C-3′ and antisense: 5′-GGA CTC CTT AGA CTC ACC GCT CTT-3′, *Smad1* sense: 5′-ACG GGT TCG AGA CCG TGT AT-3′ and antisense: 5′-CAT CCT GCC GGT ATT CG-3′, *Smad4* sense: 5′-TGG GTC CGT GGG TGG AAT A-3′ and antisense: 5′-GAG GTC ATC CAC ACC GAT GC-3′, *Smad5* sense: 5′-ACC GCA CAT GCC ACA AAA C-3′ and antisense: 5′-CAG GGG AAG GAG GAT AGG G-3′, and *β-actin* sense: 5′-AAG GCC AAC CGT GAA AAG AT-3′ and antisense: 5′-GTG GTA CGA CCA GAG GCA TAC-3′.

### 4.9. HPLC Analysis

The SRE and SBE were dissolved in 80% MeOH to a final concentration of 2 mg/mL and filtered through a 0.45 μm GL Chromato disk 13 A (GL Sciences, Tokyo, Japan) before HPLC. HPLC analysis were performed on an X-LC system (pump: 3185PU, degasser: 3080DG, mixer: 3180MX, column oven: 3067CO, autosampler: 3159AS, detector: 3110MD; JASCO, Tokyo, Japan). A 5C_18_-MS-II (4.6 mm i.d. ×150 mm, 5 µm) (Nacalai Tesque) was used for the chromatography at a flow rate of 1.0 mL/min and a column temperature of 40 °C. The injection volume was 5 μL. The mobile phase was composed of A (0.1% TFA aq.) and B (CH_3_CN) using a gradient ranging from 10% to 90% mobile phase B in a period time of 20 min. The detection wavelength was measured using a PDA range of 200–650 nm, and chromatograms at 210 nm for detecting several classes of compounds and 200–500 nm for detecting the characteristic UV of flavonoids were selected. Retention times of baicalin and wogonin standards (FUJIFILM Wako Pure Chemical) were as follows: baicalin: 8.12 min, wogonin: 13.32 min. All HPLC chromatograms are shown in the Supplementary Material (Figure S2).

### 4.10. Statistical Analysis

The results are expressed as mean ±SE. Statistical comparisons of different treatment groups were examined using one-way analysis of variance (ANOVA) with Dunnett’s multiple comparison test using JMP 14 (SAS Institute, Cary, NC, USA). Differences were considered significant at *p* < 0.05 and *p* < 0.01.

## 5. Conclusions

A luciferase reporter assay showed that SRE had ER transcription-promoting activity in HEK293 cells and no proliferation-promoting activity in MCF-7 breast cancer cells. The extract also enhanced the ALP activity of MC3T3-E1 osteoblast-like cells, and showed weak calcification-promoting activity. These findings were supported by the results of the gene expression analysis, which showed activation of the bone-formation-related genes *Runx2* and *Osx* and bone formation markers. The gene expression analysis revealed that SRE might promote osteogenesis via the BMP/*Smad* signaling pathway. According to the alizarin red stain assay and gene expression analysis by quantitative real-time PCR, SRE acts more strongly on osteoblasts in the early stage of differentiation than in the late stage of differentiation. On the other hand, it showed no specific effect on osteoclast differentiation. Since SRE not only has an estrogen-like effect but also has a bone-formation-promoting effect without inhibiting bone resorption, it has the potential to improve both postmenopausal complaints and the risk of osteoporosis while maintaining normal bone remodeling.

## Data Availability

Not applicable.

## Supplementary Materials

The following supporting information can be downloaded at: https://www.mdpi.com/article/10.3390/plants11162075/s1, Figure S1: Cytotoxicity of SRE on MC3T3-E1 cells. The proliferation of MC3T3-E1 cells was assessed using the MTT assay. The control contained DMSO only. Data are the mean ± SE of three independent experiments (*n* = 3); Figure S2: HPLC chromatograms of SRE and SBE. Detection: (a) 210 nm, (b) 200–500 nm.
