# Supplementary material for: Effects of a Whole Plant Extract of Scutellaria rubropunctata var. rubropunctata on Bone Metabolism with Estrogen Receptor Activation
Source: Plants (Basel). 2022 Aug 9;11(16):2075. doi: 10.3390/plants11162075 (PMC9412382; doi:10.3390/plants11162075)
Supplement: Supplementary file 1 [file plants-11-02075-s001.zip › plants-1819621-supplementary.pdf]

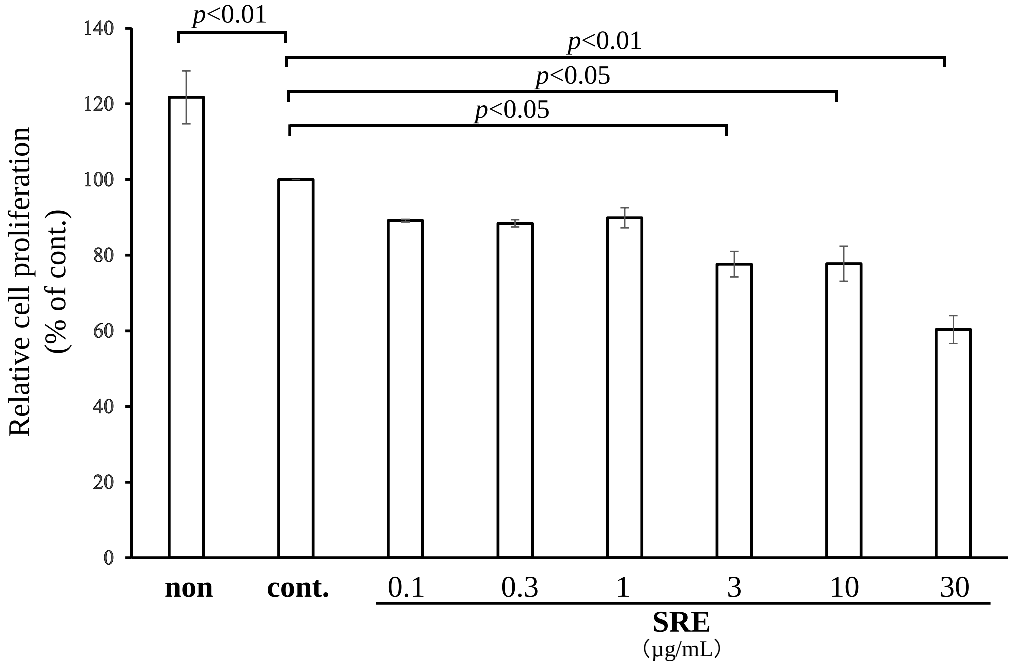

Figure S1. Cytotoxicity of SRE on MC3T3-E1 cells. Proliferation of MC3T3-E1 cells was assessed using the MTT assay. The control contained DMSO only. Data are the mean ± SE of three independent experiments (n=3).

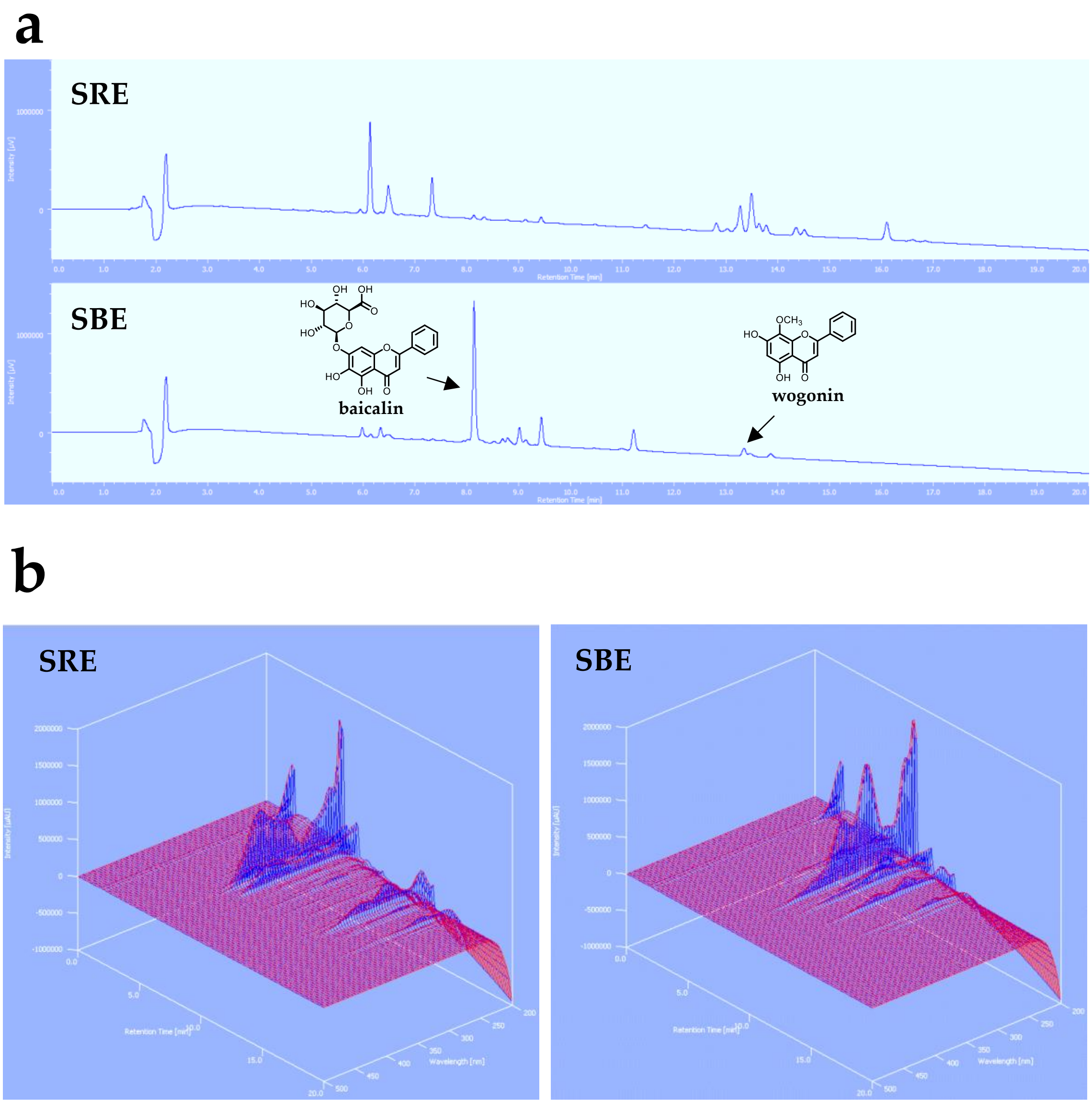

Figure S2. HPLC chromatograms of SRE and SBE. Detection: (a) 210 nm, (b) 200－500 nm.
